# Development of a Personal Guide Robot That Leads a Guest Hand-in-Hand While Keeping a Distance

**DOI:** 10.3390/s24072345

**Published:** 2024-04-07

**Authors:** Hironobu Wakabayashi, Yutaka Hiroi, Kenzaburo Miyawaki, Akinori Ito

**Affiliations:** 1 Graduate School of Robotics and Design, Osaka Institute of Technology, Osaka 530-8568, Japan; m1m22r41@st.oit.ac.jp; 2 Faculty of Robotics and Design, Osaka Institute of Technology, Osaka 530-8568, Japan; yutaka.hiroi@oit.ac.jp; 3 Faculty of Information Sciences and Technology, Osaka Institute of Technology, Hirakata 573-0196, Japan; kenzaburo.miyawaki@oit.ac.jp; 4 Graduate School of Engineering, Tohoku University, Sendai 980-8579, Japan

**Keywords:** guide robot, human–robot interaction, robot interfaces, mobile robot

## Abstract

This paper proposes a novel tour guide robot, “ASAHI ReBorn”, which can lead a guest by hand one-on-one while maintaining a proper distance from the guest. The robot uses a stretchable arm interface to hold the guest’s hand and adjusts its speed according to the guest’s pace. The robot also follows a given guide path accurately using the Robot Side method, a robot navigation method that follows a pre-defined path quickly and accurately. In addition, a control method is introduced that limits the angular velocity of the robot to avoid the robot’s quick turn while guiding the guest. We evaluated the performance and usability of the proposed robot through experiments and user studies. The tour-guiding experiment revealed that the proposed method that keeps distance between the robot and the guest using the stretchable arm enables the guests to look around the exhibits compared with the condition where the robot moved at a constant velocity.

## 1. Introduction

Robots that work instead of humans are becoming increasingly popular. A tour guide robot [1,2] is one such example. Compared with other methods [3], a robot-based tour guide has the advantage of not requiring any changes to the environment, such as landmarks [4], directional signs [5], and beacons [6]. Iio et al. reported [7] that people wanted to use the robot’s guidance again.

There are two kinds of tour guide robots. The first one is installed in a specific location and provides information using voice, display, and gestures at that location [8]. This type of robot is a kind of information kiosk [9,10], where the robot is installed at a fixed place and explains information to the visitors. Yonezawa et al. proposed a guidance system where multiple information kiosk robots work together [11]. This kind of robot is efficient when the number of points of interest (PoI) is small. When we have many PoIs, installing many robots at each PoI is not feasible. The second is a mobile robot that moves around the place, such as a museum or a campus, and leads the guests from PoI to PoI.

We can consider two cases when guiding guests: one guide leads multiple guests, and one guide leads a guest one-on-one. Guiding multiple guests is an efficient method in terms of time; however, it is difficult for a guide to match all the requests from the guests, such as keeping pace with guests and explaining objects in which the guests are interested. On the other hand, in one-on-one guiding, the robot can guide the guest at the guest’s pace, for example, by allocating time to guide to places the guest wants to take time. In addition, the guided tour has the advantage of facilitating communication with the guided tour participants. For these advantages, this study focuses on one-on-one guidance and aims to guide a single mobile robot to a destination.

This research aims to realize a guide system where a mobile robot travels along a route. A robot [12,13] that travels along a route reduces the burden on the guest to memorize the route, and the robot can also add explanations while guiding. However, as Ichihara et al. [14] point out, it is necessary to change the robot’s behavior in response to the guest’s movements, and the robot must constantly monitor the guest’s condition. In addition, according to Shiomi et al.’s report, the guest sometimes leaves the robot during guidance [15], so it is necessary to devise a way to have the guided robot always follow the guest.

One of the possible ways to achieve this is to exploit physical interaction with the guest. Robots that guide a visitor through physical contact between the robot and the visitor, such as LIGHBOT [16] or the suitcase-type robot [17], are intended to help visually impaired people. This interaction between humans occurs in a close relationship, and research on hand-holding human–robot interaction has been conducted, such as Hasegawa et al.’s Mako-no-te [18]; however, its application to tour-guiding has been limited.

Therefore, this study aims to develop a personal tour guide robot that can lead the way by pulling hands while maintaining a good distance from a person. We have developed a robot, “ASAHI ReBorn”, based on the daily life support robot ASAHI [19]. ASAHI is a multi-purpose robotic platform that has a mobile base and a small communication robot (the robot avatar). ASAHI ReBorn’s unique feature is that its robot avatar has an extendable arm. When it leads a guest, the guest holds the robot’s hand. The tension of the arm and the human–robot distance are beneficial to control the robot’s velocity so that it moves at the pace of the guest.

The contributions of the current work are as follows.

- The guide robot could hold hands with the guided person and lead them to the final destination.
- We realized distance control of the robot to move while keeping a good distance from the guest.

In the following sections, we explain how these contributions are achieved.

## 2. Related Work

Many tour guide robots have been developed so far. Development of such robots began in the late 1990s [1,20]. The early works focused on building a robotic system that recognizes the environment, plans the navigation path, and interacts with the guests. RHINO [20,21] was a mobile robot for a museum tour guide that could make a map of the museum, avoid collisions, and interact briefly with guests. It worked in the Deutsches Museum Bonn. MINERVA [1] was a robot with similar functionalities that worked in the Smithsonian Museum. Schraft et al. developed a tour guide robot [22] using Care-O-Bot, which could communicate with the guests for entertainment. This robot worked in the Museum für Kommunikation, Berlin. Urbano [23] was a tour guide mobile robot with arms and a face with expressions, and it could interact with visitors and guide them in a museum.

After these robots, many improvements have been made; in particular, many research studies have focused on the interaction between the robot and guests. For example, Kim et al. developed a tour guide robot, Jinny [24], which behaved autonomously and chose its navigation strategy according to the robot’s status. Shiomi et al. investigated how the interaction between a robot and guests increased the guest’s impression of the museum [25]. Kuno et al. investigated the effect of the robot’s gesture on the guest’s impression [26]. They found that the robot’s head movement could enhance the visitor’s engagement with the robot. Díaz et al. investigated the group interaction using the humanoid robot REEM [27]. They installed the robot at the CosmoCaixa Museum and observed interactions between it and visitor groups. Ghosh and Kuzuoka investigated how the robot’s verbal and nonverbal behavior affected the guests’ interest in the exhibition [28]. Karreman et al. investigated how the guests interact with the guide-tour robot FROG [29]. They carried out an operation experiment in Royal Alcázar in Seville, Spain, and analyzed the behavior and impression of spontaneous and invited visitors. Rashed et al. developed a robot that estimated the guest’s intentions from their behavior and initiated the guide [30].

In this decade, along with the development of artificial intelligence technologies, many component technologies for this kind of robot have become easier and more accurate, such as simultaneous localization and mapping (SLAM) [31], path planning [32], speech recognition [33], speech synthesis [34], face recognition [35], gesture generation [36], and dialogue management [37]. These developments make the development of such robots relatively easy compared with the situation a decade ago.

Because the technologies have matured, the research focus has changed to establishing relationships between robots and visitors. Gehle et al. investigated how to determine the timing for a robot to establish interaction with a visitor [38]. Del Duchetto et al. developed a tour guide robot, Lindsay, which worked in a museum and guided the visitors [39]. They investigated the visitors’ engagement with the tour guide robot. Iio et al. developed a museum guide robot that identified individual guest, called their name, and made friendly interactions [7]. Vásquez et al. developed a tour guide robot [40] that expresses emotion using facial expressions. They investigated how the use of facial expressions affects the visitor’s impression of the robot.

As explained above, recent research on tour guide robots mainly focuses on the interaction between the robot and visitors. Multimodal information such as speech, face, and gesture is used for communication channels, and their effect is investigated. However, none of these works of literature assume that the robot and visitor have physical contact.

When two persons walk hand-in-hand, it is known that their walking pace synchronizes [41,42]. This fact suggests that a robot and a guest could mutually control the walking pace if they held hands. Hasegawa et al. developed a mobile robot, Mako-no-te [18], which moves with a user hand-in-hand. Similarly, Kochigami et al. [43] developed a method to control a robot moving hand-in-hand with a child. They used the humanoid robot Pepper (https://www.aldebaran.com/en/pepper, accessed on 30 March 2024) as a platform and succeeded in controlling the robot using the pulling force of the hand. From a psychological aspect, it is known that walking hand-in-hand improves the user’s impression of the robot [44]. Therefore, applying the “hand-in-hand” movement to a tour guide robot can be possible. Nakane et al. developed a robot guidance system that holds the guest’s hand while guiding [45].

Our work aims to let the guest decide how to look around the facility by communicating with the robot by holding its hand. During a tour, guests may take their time to see certain exhibits or skip others; however, a tour guide with a steady pace does not give guests that freedom. Thus, we designed a robot with an extendable hand, and the guest held the hand while being guided. The proposed method enables the tour guide robot to match the guest’s pace by communicating with the tactile information obtained from the hand.

## 3. Design of the Tour Guide Robot

### 3.1. Requirements for the Tour Guide Robot

We defined the following two requirements to achieve the objectives.

- To accurately guide the guest along the pre-defined route from the start to the end.
- Move with the guest at the guest’s walking pace.

If the first requirement is not achieved, the robot may deviate from the guide route, which may cause passing by the PoIs or collide with walls or other objects in a narrow space such as a corridor. Therefore, we need a method to move along a given path accurately. In addition, the robot must guide the guest to the destination along the path. To do that, the robot must interact with the guest so that they are within a certain distance.

Even if only the first requirement is achieved, the robot will continue to lead the guest at a constant speed when the guest stops in the vicinity of an exhibit of interest to take his/her time to look at it. As Bönsch et al. pointed out [46], the robot needs to lead the guided tour according to the pace of the guest, which is the second requirement.

### 3.2. Design Concept

Next, we describe the design concept of the tour guide robot that satisfies the above requirements. Regarding the first requirement, the robot must move accurately along the guide route. The robot can stop when turning and rotate on the spot to minimize errors with the route to minimize the error from the route. However, according to Reinhardt et al. [47], a person following a robot does not like the robot to make small turns. Therefore, the robot needs to move along the route smoothly and quickly. We proposed the Robot Side method [48] to achieve these requirements.

In addition, it is not guaranteed that the guest will always follow the robot if the robot only leads the way. One way to ensure this is to physically restrain the guest, such as letting the guest hold the robot’s arm. However, restraining the guest with a rigid arm can be dangerous, as the guest can be swung around when the robot turns. Moreover, if the guest is restrained tightly, the guest must strictly follow the robot’s movement, which violates the second requirement. Therefore, we installed an extendable arm for the robot, and the guest held the hand to move with the robot.

To satisfy the second requirement, the robot must grasp the guest’s pace and adjust its velocity accordingly. For example, if the guest stops near an exhibit to look at something interesting, the robot may also stop and wait. To achieve this, we focused on the distance between the guest and the robot. When the guest moves slowly, the distance increases; the distance decreases when the guest follows the robot. Therefore, we implement the “pacing control” [49], a speed control method that maintains pacing by adjusting the robot’s speed.

**Figure 1:**
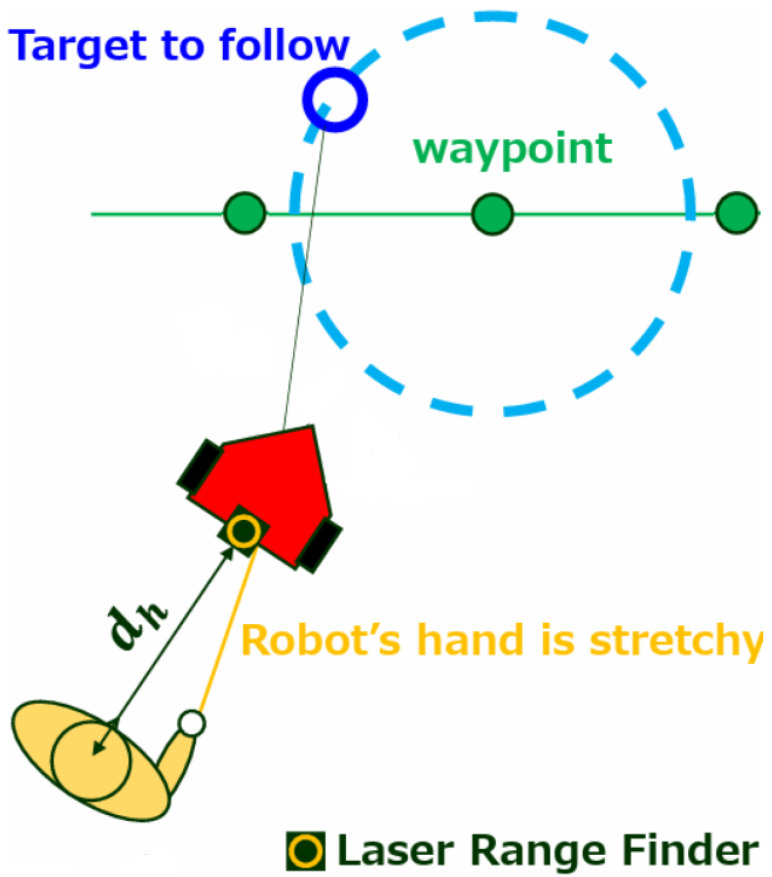
The tour guide robot “ASAHI ReBorn”. This robot moves along the guide route (waypoints) using the Robot Side method (Figure 2) while the guest holds the robot’s hand. The robot controls its velocity according to the distance to the guest.

We developed a tour guide robot to satisfy the two requirements. Figure 1 shows an overview of the robot and its behavior. This robot follows the guide route using the Robot Side method and guides the guest by interacting them using an extendable string arm. The pacing control method adjusts the robot’s speed according to the guest’s pace.

### 3.3. The Robot Side Method

**Figure 2:**
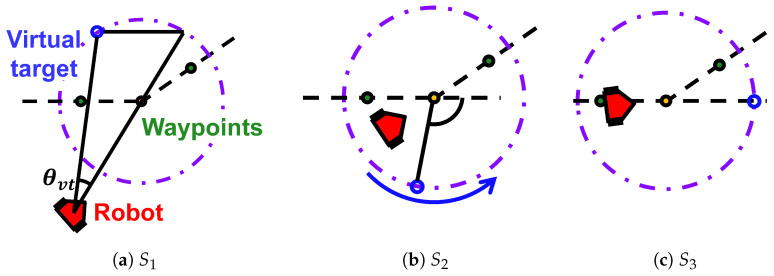
Path following using the Robot Side method. $S_{1},S_{2}$, and $S_{3}$ denote the three states of the method. (**a**) When off-path, the robot quickly returns by targeting a point on a virtual circle. (**b**) When the robot approaches the path, it avoids overshooting by moving the target to the other side of the circle and then moving it forward. (**c**) When the robot is near the path, it maintains a stable course and attitude angle alongside the path.

We exploit the Robot Side method [48] that enables a robot to follow a given route accurately with a small overshoot. As shown in Figure 2, the Robot Side method has three states determined by the positional relationship between the robot and the path. The robot follows the waypoints on the route path. Instead of simply following the waypoints, the Robot Side method assumes a virtual target on a circle around the waypoint. The position of the virtual target depends on the current state. Then, the robot moves toward the virtual target instead of the waypoint to realize a faster path following. Figure 2a shows a situation where the robot and the path are far apart, and the robot quickly moves to the path by setting the virtual target. Figure 2b shows the robot approaching the path. The virtual target is lowered vertically and then moved in a circular arc to correct the robot’s attitude angle. Figure 2c shows the robot tracking near the path, and the virtual target is fixed on the path for stable tracking.

After a virtual target is set, the robot controls the velocity to move toward this virtual target. The translational velocity is an arbitrary positive value. The angular velocity is the value shown in Equation (1), and we control the angular velocity so that $\theta_{vt}$ gets small. This study used the constant values $K_{\theta }=0.2$ and $K_{\dot{\theta }}=0.01$. $d_{t}$ is the tread width.

(1) $$\omega_{pd}=\frac{2\left(K_{\theta }\theta_{vt}+K_{\dot{\theta }}{\dot{\theta }}_{vt}\right)}{d_{t}}$$

The Robot Side method performs better (with a small fluctuation from the guide route) than Pure Pursuit, another path-following method [48].

### 3.4. Robot Avatar with Extendable Hand

To constrain the distance between the robot and the guest, we devised an idea where the guest holds the robot’s hand while being guided. Holding one’s hand is an interaction seen in close relationships between people. Hasegawa and Okada developed Mako-no-te [18], a robot with an arm that moves with a human side-by-side while the human holds the hand of the robot. Holding a robot’s hand is also considered an extension of the guiding function, such as when the guest pulls the robot’s hand to convey his/her intention to stop.

We need to consider the range of motion and safety of the robot’s hand mechanism. Specifically, the guest must be able to continue holding the robot’s hand even if the distance to the robot changes. Moreover, the arm should be safe and not harm the guest or other objects in the environment. Therefore, in this research, we develop an “extendable hand” that attaches to the arm part of the robot. The extendable hand is a mechanism in which a hand (a ball of styrene foam) attached to the end of a string is wound by a pulley, and a person grips the robot’s hand part.

It is also important for the robot to communicate with the guest through voice and gestures, such as explaining the facilities, instead of silently pulling the guest’s hand. Therefore, we consider incorporating this extendable hand inside the Robot Avatar [50], a communication robot developed by this research group. The amount of rotation of the pulley enables the robot to know the length of the arm (string) being pulled so that it can interact with the guest by pulling back in response to the amount of pull.

**Figure 3:**
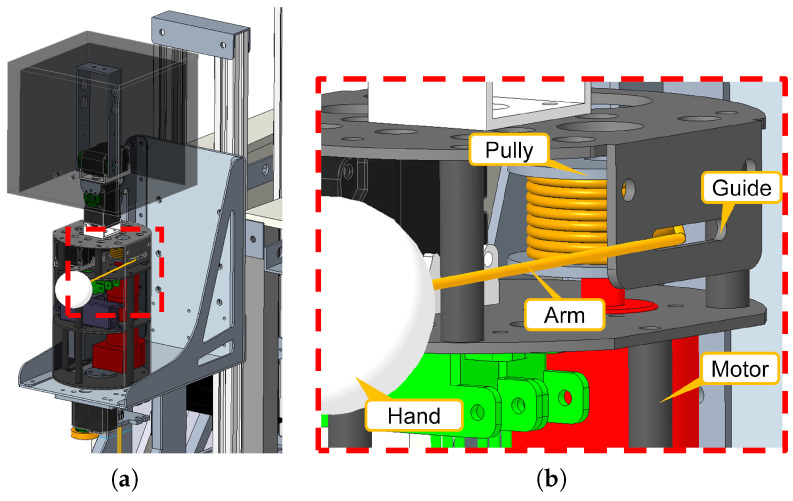
The mechanism of a robot avatar with an extendable arm. The arm’s string is wound around a pulley attached to a motor unit. (**a**) Overall view. (**b**) Close view.

**Figure 4:**
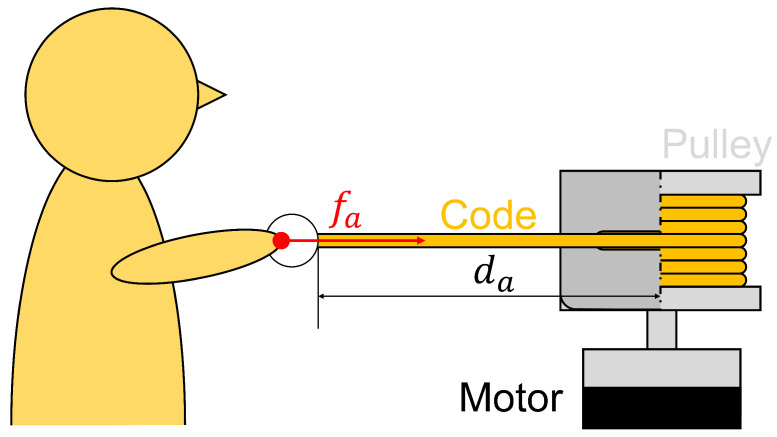
The tension $f_{a}$ and the pulling distance $d_{a}$. The tension is proportional to the distance, i.e., $f_{a}=K_{f}d_{a}$.

Specifically, as shown in Figure 3a, the left arm of the robot avatar is an extendable and retractable hand. As shown in Figure 3b, the arm (string) is wound around a pulley attached to the shaft of a motor unit (DC motor: RE25, gear head: GP26A, encoder: HEDL5540 (Maxon International Ltd., Sachseln, Switzerland)). When a person pulls this hand through the guide, the arm pulls back with force $f_{a}$ corresponding to the amount $d_{a}$ pulled, as shown in Figure 4. $f_{a}$ is given by Equation (2), where $K_{f}$ is the stiffness; in this study, we used $K_{f}=5.77$. The maximum pull force is 3.0 [N], and the minimum pull force is 0 [N].

(2) $$f_{a}=K_{f}d_{a}$$

### 3.5. Controlling Human-Robot Distance by the Robot’s Speed

**Figure 5:**
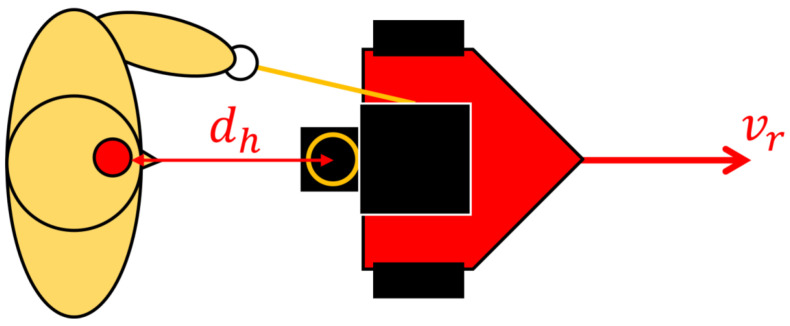
The robot’s translational speed $v_{r}$ and the human–robot distance $d_{h}$. $v_{r}$ is controlled so that $d_{h}$ becomes a pre-defined distance $D_{\max}$, i.e., $v_{r}=K_{p}\left(D_{\max}-d_{h}\right)$.

Fujiwara et al. focused on the distance $d_{h}$ between the guest and the robot. They developed “pacing control” [49], in which the robot waits for the guided subject to approach by decreasing its speed when the distance is large and moving faster by increasing its speed when the distance is small. Figure 5 shows the robot’s moving direction and distance. Specifically, the robot moves at a velocity proportional to the difference between the maximum allowable distance $D_{\max}$ and $d_{h}$.

(3) $$v_{r}=K_{p}\left(D_{\max}-d_{h}\right)$$

In this study, the maximum length of the outstretched hand $D_{\max}$ is set to 1.35 [m], and the constant $K_{p}$ is set to 0.86. The distance $d_{h}$ is calculated by the LRF-based person detection method [51]. As explained in Appendix A, this control is stable as long as $K_{p}>0$.

The handrail-moving robot developed by Fujiwara et al. considered only a straight path on the handrail, so only the straight-line component of the robot was considered, as in Equation (3). However, in this research, since the robot guides in a planar environment, it is necessary to consider the turning component as well, depending on the distance from the person. Therefore, this research considered increasing or decreasing the maximum magnitude of the robot’s angular velocity $\omega_{r}$ that can be output according to the increase or decrease of the robot’s translation velocity $v_{r}$. Equation (4) is set up so that if the current output $v_{r}$ is the maximum value $V_{\max}$, the angular velocity can also be output up to the maximum value $\Omega_{\max}$. If the translational velocity is 0, the angular velocity is also 0. In the later experiment, we used $V_{\max}=0.45$ [m/s] and $\Omega_{\max}=100$ [deg/s].

(4) $$\omega_{r}=\mathrm{sign}\left(\omega_{pd}\right)\min\left(|\omega_{pd}|,\frac{v_{r}\Omega_{\max}}{V_{\max}}\right)$$

## 4. Implementation of the Robot “ASAHI ReBorn”

In this section, we describe the implementation of the tour guide robot, which we named “ASAHI ReBorn”.

### 4.1. The System

**Figure 6:**
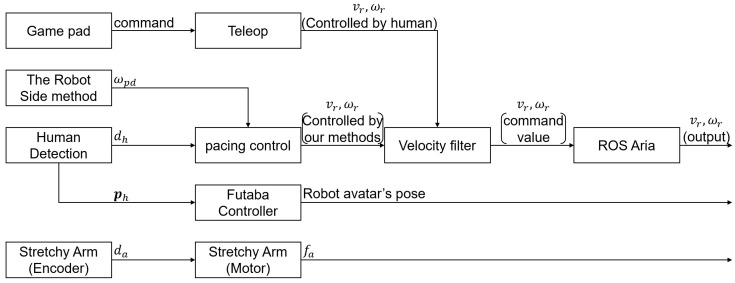
The block diagram of the system. The control processes for moving the base (the upper blocks) and the arm (the lower blocks) work independently.

Figure 6 shows the block diagram of the robot system. As shown in the figure, the control processes for moving base (the upper blocks) and that for the arm (the lower blocks) work independently. The Robot Side block determines the angular velocity $\omega_{pd}$, and the Human Detection block measures the distance to the human $d_{h}$ and the position of the human $p_{h}$. Then, the pacing control block receives $\omega_{pd}$ and $d_{h}$, and determines $v_{r}$ and $\omega_{r}$ according to Equations (3) and (4). The human position $p_{h}$ is used to control the robot avatar to face the guest. The gamepad is used to manually control the robot in case of emergency.

### 4.2. The Hardware

This section describes the development of the hardware. First, we need a mechanism to move in a real environment. Therefore, we will develop the robot based on ASAHI [52], a daily life support robot developed by our research group. Next, we need a robot avatar (Section 3.4) that connects hands with the guest. We installed the robot avatar so that it faces the guest, based on the result by Shiomi et al. [15] that a robot can attract the guest’s interest by facing the guest. The head height of the robot avatar is the same as that of the handrail-moving robot [49].

**Figure 7:**
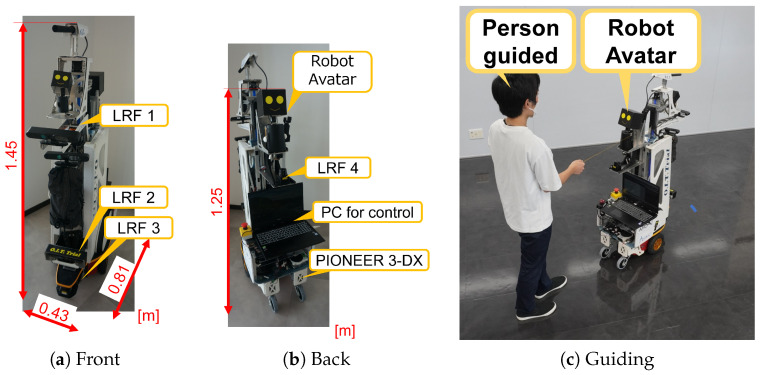
The tour guide robot ASAHI ReBorn. It has two robot avatars at the front and back of the body (**a**,**b**). It also has four LRFs, where LRF2 is used for navigation, and LRF4 is used for measuring the distance to the guest (LRF1 and LRF3 are not used in the experiments in this paper). As shown in (**c**), the guest pulls the hand (string) of the robot avatar mounted on the back while being guided.

Figure 7 shows the appearance of ASAHI ReBorn, a tour guide robot with a robot avatar mounted on ASAHI, and how ASAHI ReBorn guides the user. Figure 7a shows the appearance of ASAHI ReBorn viewed from the front (direction of travel). Figure 7b is a view from the rear (the direction where the guest is located), and Figure 7c shows ASAHI ReBorn leading a guided tour while pulling the hand of the guest.

ASAHI ReBorn is based on a Pioneer 3DX (Adept Technology, Inc., Amherst, NH, USA), an opposing two-wheeled mobile robot with a maximum translational velocity of 0.75 [m/s], a maximum angular velocity of 100 [deg/s], an acceleration of 0.30 [m/s^2^], and an angular acceleration of 96 [deg/s^2^]. This study set the maximum translational velocity to 0.45 [m/s]. The robot dimensions are 0.43×0.81×1.45 [m] (WDH), the tread width is 0.35 [m], and the mass is 29 [kg]. The PC controlling ASAHI ReBorn is DAIV 19115N-CLR (Mouse Computer, Tokyo, Japan; CPU: Intel(R) Core^TM^ i7-9750H CPU @ 2.60 GHz, Memory: 32.0 GB, OS: Windows 10). ASAHI ReBorn is equipped with four two-dimensional LRFs (Laser Range Finders). The front LRFs (LRF1-3) are UTM-30LX (Hokuyo Automatic Co. Ltd., Osaka, Japan; range: 30 [m], 270 [deg]), which are used for self-position estimation by ROS (Robot Operation System) amcl (Adaptive Monte Carlo Localization) (https://wiki.ros.org/amcl, accessed on 30 March 2024) and detection of obstacles/opponents in the direction of travel. The rear LRF is the URG-04LX-UG01 (Hokuyo Automatic Co. Ltd., Osaka, Japan; range: 4 [m], 240 [deg]), which is used to detect the guest.

### 4.3. The Software

**Figure 8:**
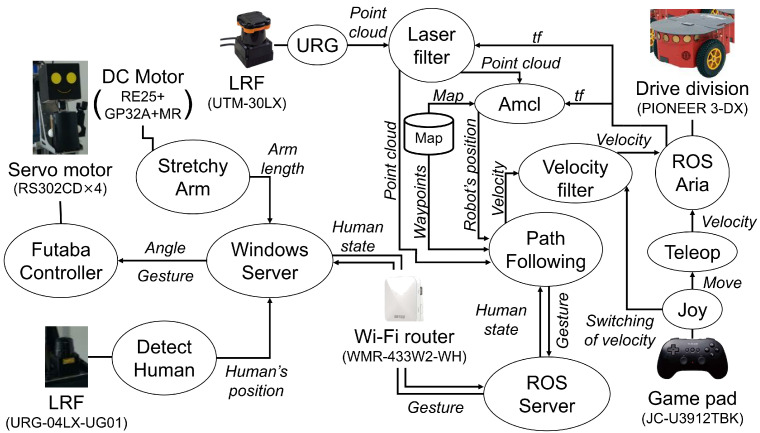
System configuration of ASAHI ReBorn. The figure’s ovals denote ROS modules. The robot avatar and arm are controlled on the Windows server, which communicates to the ROS server on Ubuntu on VMWare. The SLAM task and the mobile base control work on Ubuntu.

In the implementation, we focused on integrating the ROS node with the Windows 11 software. This mechanism enabled us to effectively use our existing Windows software assets. We used ROS melodic [53] 1.14.13 to control the body of ASAHI, including the Robot Side method. We also used Linux (Ubuntu 18.04.08 LTS) using VMware Workstation 16 Pro (16.2.4) on Windows 11 OS for the interaction part of the robot avatar. The processes of the two operating systems are interconnected using inet sockets via a WiFi router (WMR-433W2-WH, Buffalo Inc., Nagoya, Japan). The system configuration is shown in Figure 8. A Windows Server manages the robot avatar, sending information to the ROS server from three nodes processed in parallel: the “Detect Human” node (LRF detects the guest), the “Futaba Controller” node (controls the robot avatar’s posture), and the “Stretchy Arm” node (controls the hand’s extension force). Besides, ROS handles these nodes in parallel: the “Path Following” node (path following), “Joy” node (manual control command reception), “ROS Aria” node (control of Pioneer 3DX), “Velocity Filter” node (speed control including switching between autonomous movement and manual control), “Amcl” node (self-position estimation using amcl), and the “Laser Filter” node (filtering of LRF-acquired points (https://wiki.ros.org/laser_filters, accessed on 31 March 2024). The path following is performed by referring to a map (environmental map created in advance by gmapping (https://wiki.ros.org/gmapping, accessed on 31 March 2024) and manually placed waypoints).

**Figure 9:**
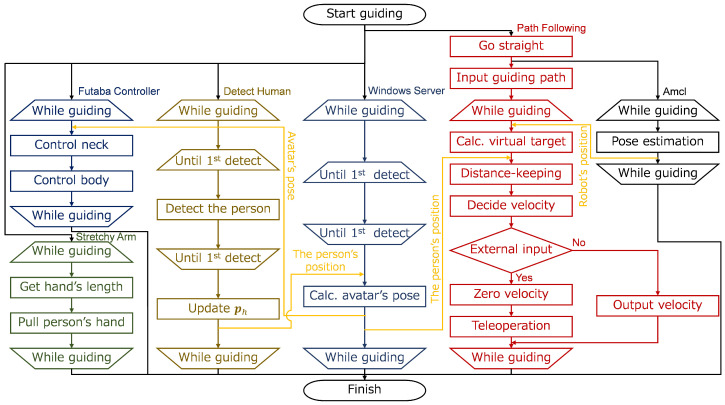
The control flow of ASAHI ReBorn. Six processes (Futaba Controller, Stretchy Arm, Detect Human, Windows Server, Path Following, and Amcl) run in parallel, exchanging data using sockets. $p_{h}$ in the figure is the center coordinate of the guest measured by the LRF [51].

The system shown in Figure 8 is controlled by the flow shown in Figure 9. The yellow lines represent the data flow. First, after the guidance starts, each node runs in parallel. The Windows server controls the avatar’s posture so that it faces the guest. Detect Human node detects a person at a pre-defined initial position, and the detection continues to update the guest’s position. Next, the nodes’ processing at ROS is as follows. After the guidance starts, the Path Following node makes the robot move 0.50 [m] straight ahead. After that, Amcl starts estimating the self-position, and the estimation continues until the end of guidance.

The Path Following loads the map and the guidance route. After that, it calculates a virtual target according to the positional relationship between the updated self-position and the path. The system calculates a speed command value to move toward the calculated virtual target using the guest’s position and posture received from the Windows Server. However, after the pacing control calculates the speed, the speed command value is determined according to the hardware limit value. The speed command is determined by the presence or absence of external input from the Joy; if there is an input from the Joy, the speed is set to 0, and the system can switch to manual control.

### 4.4. Route Guidance Flow

**Figure 10:**
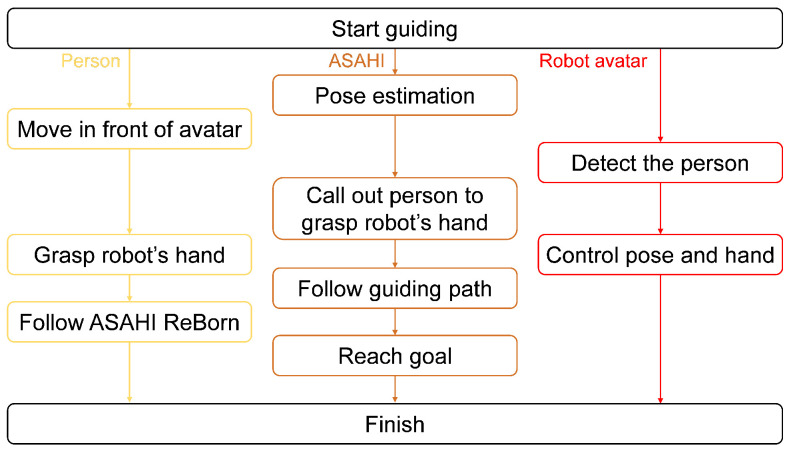
Control flow of tour guidance by ASAHI ReBorn. This figure shows the total behavior of the guest (person), the robot (ASAHI) and the robot avatar.

Figure 10 shows the flow of the tour guidance by ASAHI ReBorn. Before the guidance starts, ASAHI ReBorn estimates its own position. At the beginning of guidance, the guest moves behind ASAHI ReBorn (i.e., in front of the robot avatar). The robot avatar then detects the guest using LRF and turns toward the guest. The guest is prompted to grasp the robot avatar’s stretching hand, and when the guest pulls on the hand, the hand is pulled back with a force corresponding to the amount of the pull. While holding the hand, ASAHI begins to follow the guided path. The guest follows behind the ASAHI ReBorn, and the guidance ends when the ASAHI ReBorn reaches the target point.

In the current implementation, a human operator brings the guest by ASAHI ReBorn and confirms whether they grasp the ASAHI’s hand. Moreover, when the robot guides the guest, the operator walks with it to manually control it in case of emergency.

## 5. Simulation and Real Experiments

### 5.1. Simulation of the Distance Control with the Robot Side Method

In the previous verification of the tracking performance of the Robot Side method [48], all the experiments were conducted under the condition of constant speed. In this section, we verify by simulation whether the speed change by the distance control affects the tracking performance of the Robot Side method. If the distance control does not affect the tracking performance, we can combine it with the Robot Side without any problem.

**Figure 11:**
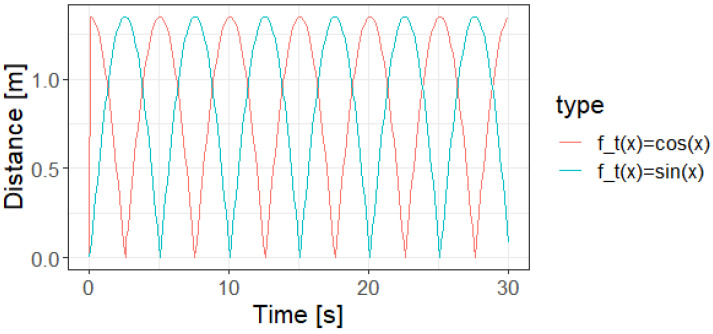
Temporal change of $d_{h}$. Two patterns of $d_{h}$, $D_{\max}\left|\cos\left(0.2\pi t\right)\right|$ and $D_{\max}\left|\sin\left(0.2\pi t\right)\right|$, were tested.

We tested the distance control by changing the value of $K_{p}$ from 0.4 to 1.6. The distance to the guest $d_{h}$ was given by Equation (5). Here, the function $f_{t}\left(x\right)$ is either sin(x) or cos(x). Figure 11 shows the temporal change of $d_{h}$. Note that $d_{h}$ could be zero in this simulation, which never happens in a real situation. We evaluated ten conditions (five parameters and two functions) and one condition where the robot simply follows the given path at a constant speed without leading a guest. We used Stage simulator (version 4.3.0), a simple robot simulator for ROS (http://wiki.ros.org/stage, accessed on 30 March 2024), for the simulation.

(5) $$d_{h}=D_{\max}\left|f_{t}\left(0.2\pi t\right)\right|$$

**Figure 12:**
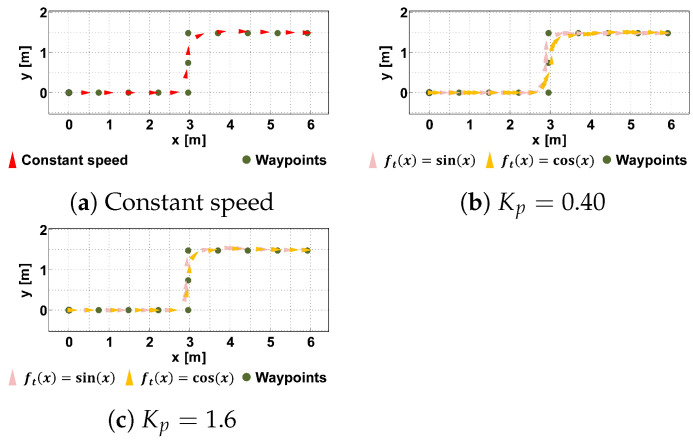
Example trajectories under each condition. (**a**) shows the trajectories of the robot without speed control (i.e., constant $v_{r}$). (**b**,**c**) are trajectories with different values of $K_{p}$.

The experimental path was a step-like path with waypoints at 0.74 [m] intervals. The robot’s maximum translational and angular velocities were set to 0.60 [m/s] and 100 [deg/s], respectively. Figure 12 shows examples of tracking results under each condition. The green circles indicate the waypoints and the triangles indicate the robot’s position and orientation. The size of the virtual robot is 0.7×0.35×1.12 (DWH) [m], and the radius of the virtual circle is 1.0 [m].

**Figure 13:**
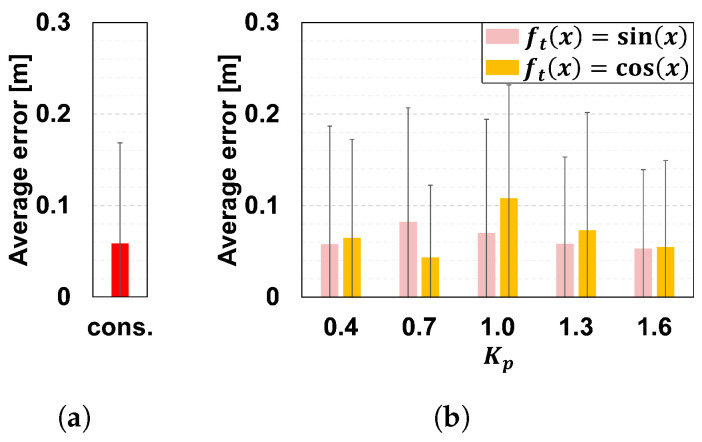
Mean squared error between the robot’s trajectory and the guidance path, and its standard deviation. (**a**) Constant speed. (**b**) Distance control.

Figure 13 shows the errors between the guidance path and the robot’s trajectory under each condition, which indicates how accurately the robot followed the path. As in [48], the error was defined as the distance between the robot and the straight line connecting the target waypoint and the previous waypoint at each time. As shown in the Figure, the difference between the error with distance control and that at a constant speed was at most 0.05 [m].

Based on the results, we concluded that the trajectory in Figure 12 and the errors in Figure 13 were not affected by the presence or absence of the distance control or its parameters. We set the parameter $K_{p}=0.86$ to vary the speed within a distance where the guest does not collide with the robot even if he/she extends his/her arm.

### 5.2. Implementation and Verification of the Distance Control on ASAHI ReBorn

In this experiment, we implemented the distance control on ASAHI ReBorn to verify whether the robot could move along a route while pulling the guided person’s hand at a speed determined by the guest’s position obtained from the LRF.

**Figure 14:**
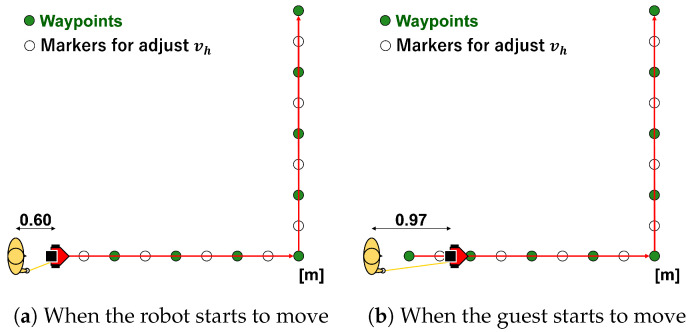
The guide route with a bend and the positions of the robot and the guest. (**a**) shows the initial positions of the guest and the robot. At first, the robot starts to move and the guest stays at the initial position until $d_{h}$ becomes 0.97 [m]. (**b**) shows the positions when the guest starts to move.

As shown in Figure 14a, ASAHI ReBorn starts moving from a distance of 0.60 [m] from the guided person. When the distance between the guest and ASAHI ReBorn reaches 0.97 [m], as shown in Figure 14b, the subject starts moving at his/her speed. We marked every 0.37 [m] on the trajectory to control the walking speed of the guest. The guests walked at a constant speed by stepping on the floor markers in time with the metronome.

We prepared three conditions for the guest’s walking conditions:

1. Walk at 0.20 [m/s] constantly from the start to the endpoint;
2. Walk at 0.45 [m/s] constantly from the start to the endpoint;
3. Walk at 0.45 [m/s], stop at the corner, then walk at 0.45 [m/s] again to the endpoint.

In addition, we examined two conditions on the angular velocity control: a condition that uses Equation (4) to determine the angular velocity $\omega_{r}$, and that without Equation (4), i.e., $\omega_{r}=\omega_{pd}$. The maximum translational velocity and angular velocity of the ASAHI ReBorn were set to 0.45 [m/s] and 100 [deg/s], respectively.

**Figure 15:**
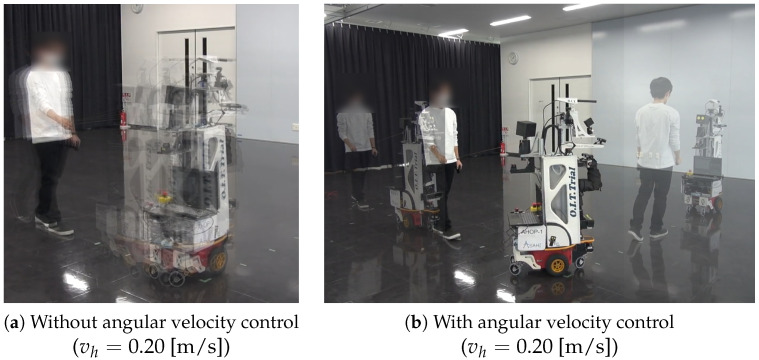
ASAHI ReBorn leads the guest using distance control. (**a**,**b**) shows the robot’s and the guest’s movement without and with the angular velocity control, respectively. In (**a**), the robot turns the corner at a right angle while it turns the corner gently in (**b**).

Figure 15 shows the robot’s movement during the experiment. These figures were created by superimposing multiple photos taken every 3 [s]. In all trials, the guests followed ASAHI ReBorn to the final destination while holding the hand of the robot avatar mounted on the ASAHI ReBorn. When ASAHI ReBorn did not perform angular velocity control using Equation (4), ASAHI ReBorn rotated in place at the corner as shown in Figure 15a under conditions 1 and 3. This behavior occurred because ASAHI ReBorn’s angular velocity did not decrease even though its translational velocity decreased according to Equations (1) and (3) outputs a value close to the maximum angular velocity when the human–robot distance increased.

Figure 15b shows the robot’s movement when ASAHI ReBorn controls the angular velocity according to Equation (4). When the guest moves away at the corner, ASAHI ReBorn adjusts its angular velocity and waits for the guided person to approach before moving and rotating. In this way, ASAHI ReBorn moved according to the pace of the guest.

**Figure 16:**
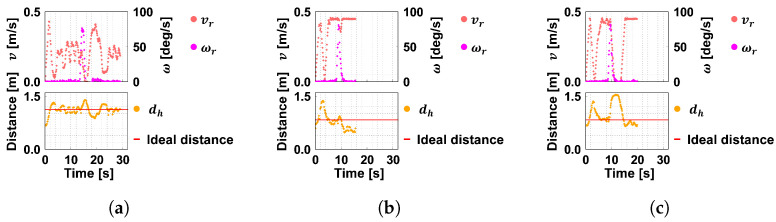
Velocity, angular velocity, and $d_{h}$ without the angular velocity control. (**a**) $v_{h}=0.20$ [m/s]. (**b**) $v_{h}=0.45$ [m/s]. (**c**) $v_{h}=0.45$ [m/s] and stop.

**Figure 17:**
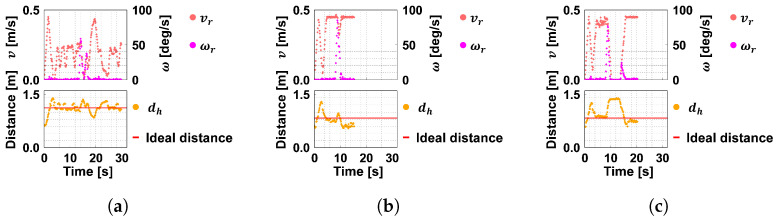
Velocity, angular velocity, and $d_{h}$ with the angular velocity control. (**a**) $v_{h}=0.20$ [m/s]. (**b**) $v_{h}=0.45$ [m/s]. (**c**) $v_{h}=0.45$ [m/s] and stop.

Figure 16 and Figure 17 show examples of the evolution of the translational velocity $v_{r}$, angular velocity $\omega_{r}$, and distance $d_{h}$. The horizontal axis shows the time transition with the start of ASAHI ReBorn’s movement set to 0 [s]. According to Equation (3), the ideal distance is the distance where $v_{r}=v_{h}$, the velocity of the robot $v_{r}$, and that of the guest $v_{h}$ coincide.

Figure 16a shows that ASAHI ReBorn moves away from the guest at the initial position and accelerates up to near 0.45 [m/s], which is the maximum translational speed; however, the distance between the guest and the robot increases because the guest moves at 0.20 [m/s]. After $d_{h}$ increases, it converges to the target distance. However, it becomes large when the guest passes the corner, and the angular velocity is about 80 [deg/s], indicating that the robot is rotating in place. On the other hand, in Figure 17a, the maximum angular velocity is only about 60 [deg/s]. The RMS (Root-Mean-Square) error between the average distance and the target value was 0.01 [m].

Figure 16b and Figure 17b are the results when the guest’s speed is 0.45 [m/s]. These results show that the angular velocity of the two conditions does not change much. This is because the robot moved at $v_{r}\approx 0.45$ [m/s] $=V_{\max}$. According to Equation (4), the angular velocity is only limited by the maximum angular velocity $\Omega_{\max}$, which is almost the same as the control without angular velocity control. In this condition, the RMS error between the average distance was 0.01 [m].

The above results show that the system was able to lead the way according to the distance to the guest by controlling the angular velocity using Equation (4). In addition, the robot could move along the guest’s pace, which satisfies one of two system requirements.

### 5.3. Experiments on Guided Tours around Exhibits

This experiment aims to verify whether distance control is appropriate for guiding guests around exhibits they see for the first time. We designed this experiment so that guests needed to spend some time at an exhibit. To achieve this, we prepared arithmetic problems as exhibits and asked the participants to tell the answers of the problems at all the exhibits.

**Figure 18:**
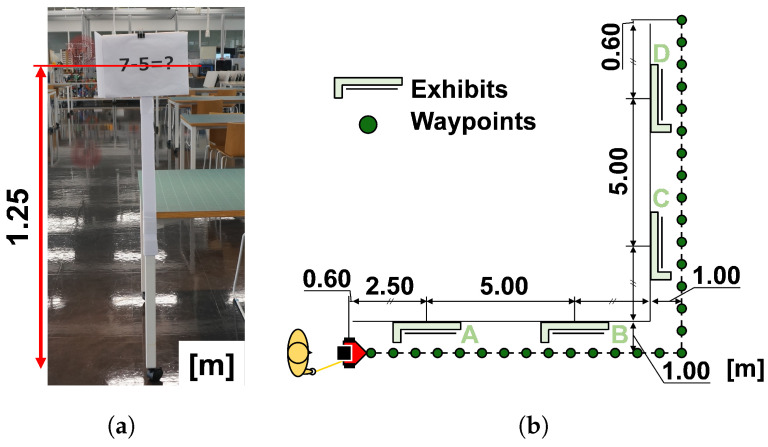
Exhibitions and the guidance path. We prepared four exhibits in a room. An exhibit has an arithmetic problem, and we asked the participants to calculate the problem of all exhibits and report them at the destination. (**a**) An example of exhibit. (**b**) The guidance path and exhibits.

Figure 18a shows the actual exhibition, a polystyrene board with a simple calculation problem printed on it and clipped to the board. Figure 18b shows the route used in the experiment and the location of the exhibits. We prepared four exhibits based on the average mental capacity [54]. Each exhibit has an arithmetic problem such as “9+2−8=?” or “5−7+6−2=?”. The guests were asked to solve and memorize the answers. After reaching the final destination, the guests answered a questionnaire about their impressions of the exhibits and the robot. The guests were given two experimental conditions: with and without distance control. Eight students from the Osaka Institute of Technology participated in the experiment and conducted it 16 times, once for each condition. All participants were familiar with robotics.

**Figure 19:**
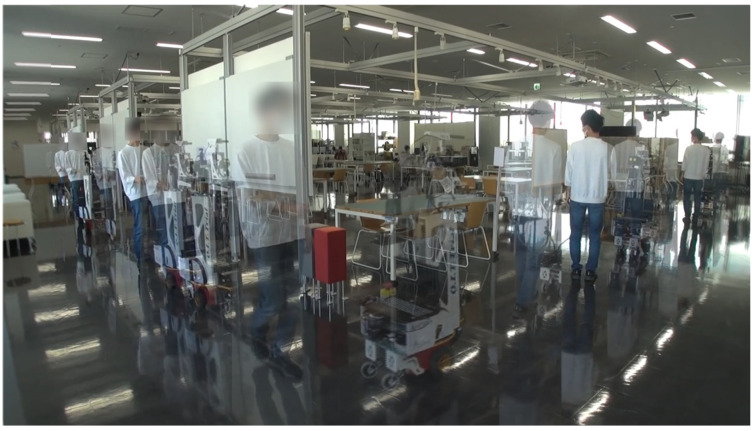
The experiment of robot tour-guiding. We can confirm that the robot and guest moved slowly at each exhibit.

Figure 19 shows that the guided participants moved after the robot while looking at the exhibits under all conditions. In the condition with no distance control, no participants stopped near an exhibit; however, in the condition with distance control, three out of eight participants stopped near one of the exhibits.

We asked the participants to answer a five-question questionnaire, and the participants answered the questions on a seven-point Likert scale. The items in the questionnaire were as follows (the names in [ ] are the labels shown in the figures):

Q1: It was easy to keep up with the robot. (1 = absolutely no, 7 = absolutely yes)
  [Q1_Easy_keepup];
Q2: I felt like I was operating the robot myself. (1 = absolutely no, 7 = absolutely yes) [Q2_Operating];
Q3: I could see the exhibit of my own volition. (1 = absolutely no, 7 = absolutely yes) [Q3_Volition];
Q4: Subjective distance to the robot (1 = too far, 7 = too near) [Q4_Distance];
Q5: Subjective speed of the robot (1 = too slow, 7 = too fast) [Q5_Speed].

In addition, we measured the following two values in the experiment:

- The time that the guided person’s face was facing the direction of the exhibits. It was measured using a web camera with a gimbal mechanism attached to the robot (Feiyu pocket, 120° angle of view) and OpenPose 1.7.0 (https://github.com/CMU-Perceptual-Computing-Lab/openpose, accessed on 30 March 2024), the human pose detection software.
- The time the participant’s movement speed decreased near an exhibit. We considered the movement slow when $v_{h}<0.20$ [m/s].

**Figure 20:**
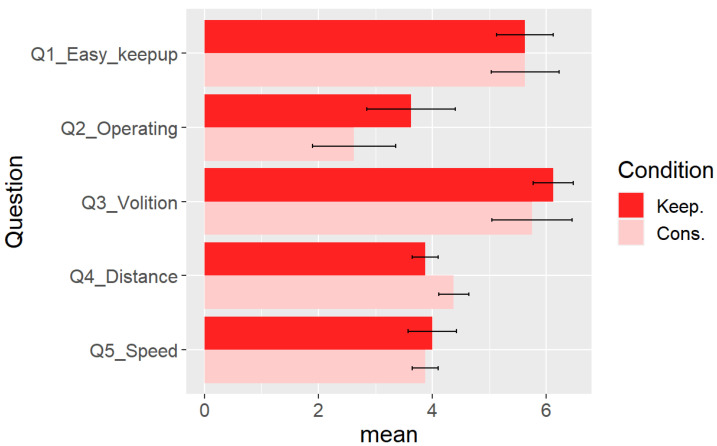
The mean values of the questionnaire. The error bars show the standard error. The labels “Keep.” and “Cons.” mean the experimental conditions with and without the distance control, respectively.

**Figure 21:**
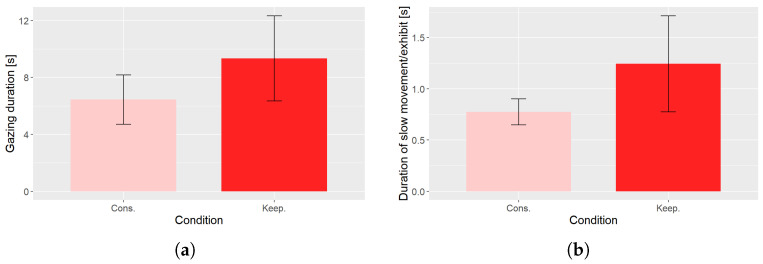
The mean values of gazing and slowly-moving time. The error bars show the standard error. The labels “Keep.” and “Cons.” mean the experimental conditions with and without the distance control, respectively. (**a**) The duration of gazing at an exhibit. (**b**) The duration of moving slowly near an exhibit.

Figure 20 shows the average of the questionnaire. The error bars of the figure show the standard error. These results suggest that the impressions were not affected by the distance control method. Figure 21 shows the difference in the duration of staying near an exhibit. Figure 21a is the duration of gazing at an exhibit, and Figure 21b is that of moving slowly near an exhibit. Both results suggest that the participants took time near an exhibit when the distance control method was used.

**Figure 22:**
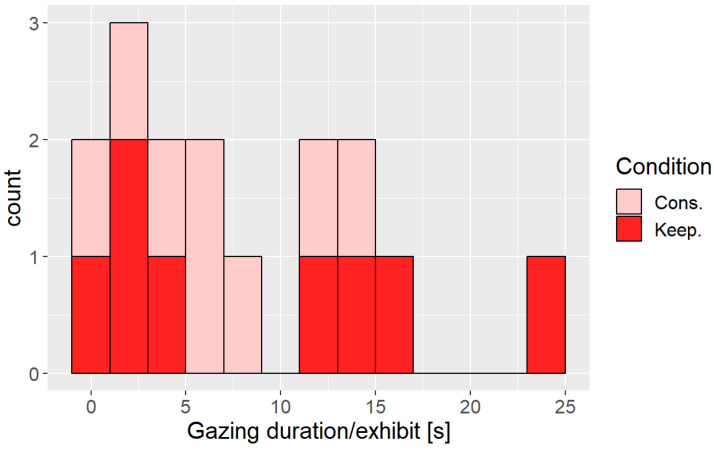
The histogram of the duration with duration of gaze at an exhibit.

Since we observed differences in the participants’ behavior near the exhibits, we investigated whether the difference in the behavior affected the participants’ impression. Figure 22 shows the duration histogram with slow movement near an exhibit. We can see that the participants with the distance control took more time than those without the distance control.

**Figure 23:**
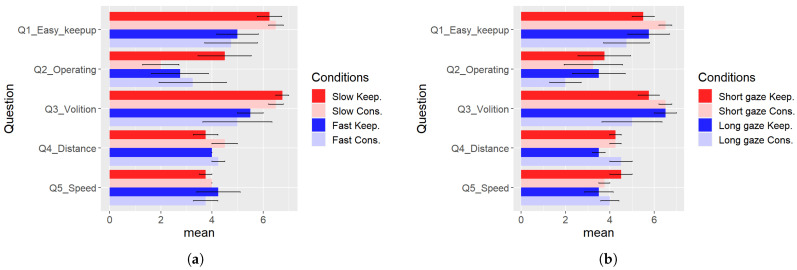
The mean values of the questionnaire, summarized group by group. The error bars show the standard error. (**a**) Summarized by Slow and Fast groups. (**b**) Summarized by Long and Short gaze groups.

We divided the participants into two groups: those who spent time near an exhibit (the “Slow” group) and the others (the “Fast” group). We chose the eight participants (four with distance control and four without one) who spent time as “Slow” and the others as “Fast.” Similarly, we divided the participants into “Long gaze” and “Short gaze” groups, each with four participants for one condition. Figure 23a shows the results of the questionnaire grouped by the control method and Slow/Fast groups. Different from Figure 20, the group-by-group analysis revealed that the participants in the Slow group felt the difference for Q2_Operating. Figure 23b shows that grouped by the control method and Long/Short gaze groups. We can see relatively large differences in Q1_Easy_keepup, Q2_Operating, and Q3_Volition of the Long gaze group.

## 6. Discussion

Ethical issues exist with using service robots in a public space such as a museum. We can consider several issues on using robots [55]; in our case, the main concern is an issue of privacy. Since the robot can record the guests’ behavior using the camera, it has a similar issue to surveillance cameras installed in public places [56]. However, our robot does not necessarily use a camera because it uses LRFs to measure guests. Thus, our robot can avoid the controversial use of cameras in public places.

**Figure 24:**
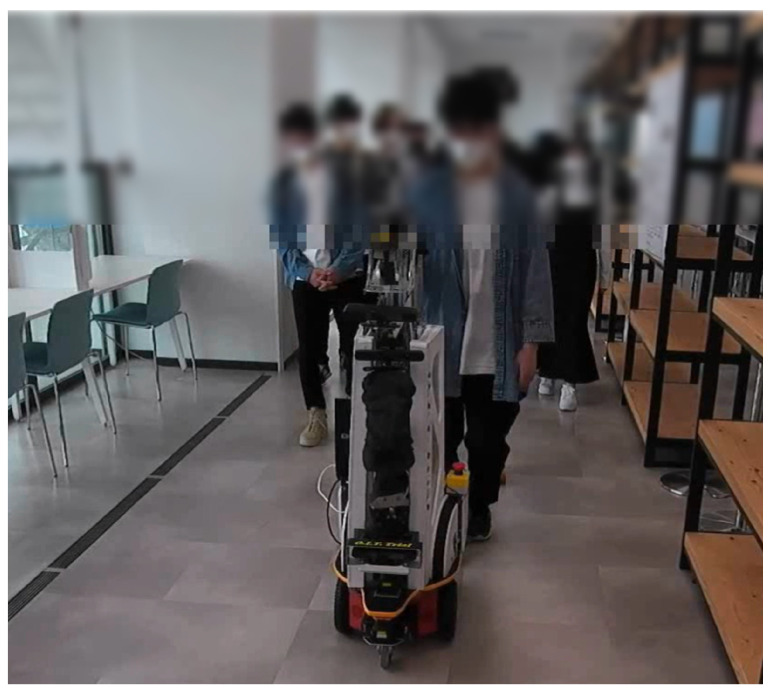
Guiding multiple guests using ASAHI ReBorn. The robot could move with the pace of the head, but it could not wait for other guests behind the head.

The limitation of this study is that it assumes one-on-one guidance, so it is not possible to move at the pace of each guided person when there are several guided persons, as shown in Figure 24. This picture was taken on 14 May 2022 at the Osaka Institute of Technology Umeda Campus, with permission of the tour group. However, the person behind the representative could not be detected due to LRF occlusion.

There are a few other limitations. First, the operator needs to help with the robot, such as finding the guest and moving back to the initial position after the guidance. When the guest comes in front of the robot avatar, the robot itself speaks to the guest to pull the robot’s hand. If the guest does not pull the robot’s hand at that time, the operator instructs the guest to do so. Moreover, if the robot is about to run into a person or other obstacles, the operator stops the robot using the gamepad. Similarly, if the robot loses its position, the operator takes it to the initial place using the gamepad.

Second, we did not implement the function to avoid obstacles and other persons to the robot. We must implement the pedestrian avoidance method [52] to realize this. Finally, the robot cannot choose the guide route dynamically according to the situation.

The various functions described above must be implemented for this robot to operate autonomously and perform guidance tasks.

## 7. Conclusions

This research developed a personal tour guide robot that leads a guest by pulling hands while keeping distance. The robot, ASAHI ReBorn, was developed by implementing the Robot Side method for following a guide route, an extendable hand as a mechanism for connecting a person and hand, and a distance control for maintaining pace with the guest. The robot was confirmed through experiments from both quantitative and subjective perspectives.

The contributions of this research are as follows.

- The guide robot could hold hands with the guided person and lead them to the final destination.
- We realized distance control of the robot to move while keeping a good distance from the guest.

The second contribution can be concluded from the results shown in Figure 17, in which $d_{h}$ converges to the “ideal distance”.

The proposed robot will have the following social and industrial impacts. First, it is expected to reduce the workload of people guiding visitors at various facilities (e.g., universities, museums, and aquariums). This will allow human guides to spend more time with visitors and provide more detailed explanations of, for example, the content of exhibits in the facility. The ideas of our robot, i.e., the robot and human can hold hands and move at the human’s pace, will increase the enjoyment of activities that involve movement, such as gait rehabilitation and walking together, not limited to guiding. Including outdoor use, there is a great demand for the task of giving directions. We believe that the system can be applied to assist visually impaired people by pulling their hands to guide them, for example, in place of guide dogs. Finally, the simple hand-holding mechanism made the robot easy to build and inexpensive.

Another possible application of this research is the rehabilitation of walking by leading a person while pulling his/her hand.

## Data Availability

The data presented in this study are available upon request from the corresponding author. Due to privacy concerns, they are not publicly available.

## Appendix A

We show that the control method shown in Equation (3) is stable. Let $x_{r}$ and $x_{h}$ be the positions of the robot and the guest, respectively. Since $v_{r}={\dot{x}}_{r}$ and $d_{h}=x_{r}-x_{h}$, Equation (3) can be rewritten as

(A1) $${\dot{x}}_{r}=K_{p}\left(D_{\max}-\left(x_{r}-x_{h}\right)\right).$$

Then, we can write the state equation as follows:

(A2) $${\dot{x}}_{r}=-K_{p}x_{r}+K_{p}\left(1\;D_{\max}\right)\left(\begin{array}{c} x_{h}\\ 1 \end{array}\right)$$

Here, $x_{r}$ is the state variable and ${\left(x_{h},1\right)}^{T}$ is the input vector. Since the coefficient of $x_{r}$ at the right hand of the equation is $-K_{p}$, its eigenvalue (i.e., $-K_{p}$ itself) is negative when $K_{p}>0$. Thus, this system is stable [57].

## References

[1] Thrun S., Bennewitz M., Burgard W., Cremers A., Dellaert F., Fox D., Hahnel D., Rosenberg C., Roy N., Schulte J. (1999). MINERVA: A second-generation museum tour-guide robot. Proceedings of the 1999 IEEE International Conference on Robotics and Automation.

[2] Peñaa K.M., Cortés B.B. (2020). GUI3DXBot: An Interactive Software Tool for a Tour-Guide Mobile Robot. Cienc. Ing. Ogran..

[3] Schwering A., Krukar J., Li R., Anacta V.J., Fuest S. (2017). Wayfinding Through Orientation. Spat. Cogn. Comput..

[4] Basiri A., Winstanley A.C., Amirian P. Landmark-based pedestrian navigation. Proceedings of the 21st GIS Research UK (GISRUK) Conference.

[5] Ko E., Kim E.Y. (2017). A vision-based wayfinding system for visually impaired people using situation awareness and activity-based instructions. Sensors.

[6] Cheraghi S.A., Namboodiri V., Walker L. GuideBeacon: Beacon-based indoor wayfinding for the blind, visually impaired, and disoriented. Proceedings of the 2017 IEEE International Conference on Pervasive Computing and Communications (PerCom).

[7] Iio T., Satake S., Kanda T., Hayashi K., Ferreri F., Hagita N. (2020). Human-Like Guide Robot that Proactively Explains Exhibits. Int. J. Soc. Robot..

[8] Kanda T., Shiomi M., Miyashita Z., Ishiguro H., Hagita N. (2010). A Communication Robot in a Shopping Mall. IEEE Trans. Robot..

[9] Lee M.K., Kiesler S., Forlizzi J. Receptionist or information kiosk: How do people talk with a robot?. Proceedings of the 2010 ACM Conference on Computer Supported Cooperative Work.

[10] Bazzano F., Lamberti F. (2018). Human-robot interfaces for interactive receptionist systems and wayfinding applications. Robotics.

[11] Yonezawa K., Suzuki Y., Ueda H., Stephanidis C., Antona M. (2013). A Map Guidance System by Multiple Dialog Robots Cooperation. Universal Access in Human-Computer Interaction. Design Methods, Tools, and Interaction Techniques for eInclusion. UAHCI 2013. Lecture Notes in Computer Science.

[12] Nguyen T.H., Tran D.N., Vo D.L., Mai V.H., Dao X.Q. (2022). AI-Powered University: Design and Deployment of Robot Assistant for Smart Universities. J. Adv. Inf. Technol..

[13] Thomas T., Doran M., Sakalaukus J. An Autonomous Campus Tour Guide Robot as a Platform for Collaborative Engineering Design. Proceedings of the 2010 Annual Conference & Exposition.

[14] Ichihara K., Hasegawa T., Yuta S., Ichikawa H., Naruse Y. (2022). Waypoint-Based Human-Tracking Navigation for Museum Guide Robot. J. Robot. Mechatronics.

[15] Shiomi M., Kanda T., Ishiguro H., Hagita N. (2010). A Larger Audience, Please!—Encouraging people to listen to a guide robot. Proceedings of the 2010 5th ACM/IEEE International Conference on Human-Robot Interaction.

[16] Tobita K., Sagayama K., Mori M., Tabuchi A. (2018). Structure and examination of the guidance robot LIGHBOT for visually impaired and elderly people. J. Robot. Mechatron..

[17] Kayukawa S., Sato D., Murata M., Ishihara T., Kosugi A., Takagi H., Morishima S., Asakawa C. (2022). How Users, Facility Managers, and Bystanders Perceive and Accept a Navigation Robot for Visually Impaired People in Public Buildings. Proceedings of the 2022 31st IEEE International Conference on Robot and Human Interactive Communication (RO-MAN).

[18] Hasegawa K., Okada M. (2019). Mako-no-te: Investigating Intersubjectivity with Side-by-Side Walking Robot. Proceedings of the 2019 7th International Conference on Human-Agent Interaction (HAI ’19).

[19] Hiroi Y., Ito A. (2013). ASAHI: OK for failure A robot for supporting daily life, equipped with a robot avatar. Proceedings of the 2013 8th ACM/IEEE International Conference on Human-Robot Interaction (HRI).

[20] Burgard W., Cremers A.B., Fox D., Hähnel D., Lakemeyer G., Schulz D., Steiner W., Thrun S. (1998). The Interactive Museum Tour-Guide Robot. Proceedings of the 1998 National Conference on Artificial Intelligence (AAAI-98).

[21] Burgard W., Cremers A.B., Fox D., Hähnel D., Lakemeyer G., Schulz D., Steiner W., Thrun S. (1999). Experiences with an interactive museum tour-guide robot. Artif. Intell..

[22] Schraft R.D., Graf B., Traub A., John D.D.I. (2001). A mobile robot platform for assistance and entertainment. Ind. Robot. Int. J..

[23] Rodriguez-Losada D., Matia F., Galan R., Hernando M., Montero J.M., Lucas J.M. (2008). Urbano, an interactive mobile tour-guide robot. Advances in Service Robotics.

[24] Kim G., Chung W., Kim K.R., Kim M., Han S., Shinn R.H. (2004). The autonomous tour-guide robot Jinny. Proceedings of the 2004 IEEE/RSJ International Conference on Intelligent Robots and Systems (IROS).

[25] Shiomi M., Kanda T., Ishiguro H., Hagita N. (2006). Interactive humanoid robots for a science museum. Proceedings of the 2006 1st ACM SIGCHI/SIGART Conference on Human-Robot Interaction.

[26] Kuno Y., Sadazuka K., Kawashima M., Yamazaki K., Yamazaki A., Kuzuoka H. (2007). Museum guide robot based on sociological interaction analysis. Proceedings of the 2007 ACM SIGCHI Conference on Human Factors in Computing Systems.

[27] Díaz-Boladeras M., Paillacho D., Angulo C., Torres O., González-Diéguez J., Albo-Canals J. (2015). Evaluating group-robot interaction in crowded public spaces: A week-long exploratory study in the wild with a humanoid robot guiding visitors through a science museum. Int. J. Humanoid Robot..

[28] Ghosh M., Kuzuoka H. (2014). An ethnomethodological study of a museum guide robot’s attempt at engagement and disengagement. J. Robot..

[29] Karreman D., Ludden G., Evers V., Tapus A., André E., Martin J.C., Ferland F., Ammi M. (2015). Visiting cultural heritage with a tour guide robot: A user evaluation study in-the-wild. Social Robotics. ICSR 2015, Paris, France, 26–30 October 2015, Lecture Notes in Computer Science.

[30] Rashed M.G., Suzuki R., Lam A., Kobayashi Y., Kuno Y. (2015). Toward museum guide robots proactively initiating interaction with humans. Proceedings of the 2015 Tenth Annual ACM/IEEE International Conference on Human-Robot Interaction Extended Abstracts.

[31] Taheri H., Xia Z.C. (2021). SLAM; definition and evolution. Eng. Appl. Artif. Intell..

[32] Aggarwal S., Kumar N. (2020). Path planning techniques for unmanned aerial vehicles: A review, solutions, and challenges. Comput. Commun..

[33] Malik M., Malik M.K., Mehmood K., Makhdoom I. (2021). Automatic speech recognition: A survey. Multimed. Tools Appl..

[34] Kaur N., Singh P. (2023). Conventional and contemporary approaches used in text to speech synthesis: A review. Artif. Intell. Rev..

[35] Kortli Y., Jridi M., Al Falou A., Atri M. (2020). Face recognition systems: A survey. Sensors.

[36] Liu Y., Mohammadi G., Song Y., Johal W. (2021). Speech-based gesture generation for robots and embodied agents: A scoping review. Proceedings of the 2021 the 9th International Conference on Human-Agent Interaction.

[37] Dai Y., Yu H., Jiang Y., Tang C., Li Y., Sun J. (2020). A survey on dialog management: Recent advances and challenges. arXiv.

[38] Gehle R., Pitsch K., Dankert T., Wrede S. (2021). How to open an interaction between robot and museum visitor? Strategies to establish a focused encounter in HRI. Proceedings of the 2017 ACM/IEEE International Conference on Human-Robot Interaction.

[39] Del Duchetto F., Baxter P., Hanheide M. (2019). Lindsey the tour guide robot-usage patterns in a museum long-term deployment. Proceedings of the 2019 28th IEEE International Conference on Robot and Human Interactive Communication (RO-MAN).

[40] Vásquez B.P.E.A., Matía F. (2020). A tour-guide robot: Moving towards interaction with humans. Eng. Appl. Artif. Intell..

[41] Roerdink M., van Ulzen N.R., de Poel H. When two become one: Spontaneous pattern formation in side-by-side and hand-in-hand walking. Proceedings of the Joint Action Meeting.

[42] Sylos-Labini F., d’Avella A., Lacquaniti F., Ivanenko Y. (2018). Human-Human Interaction Forces and Interlimb Coordination during Side-by-Side Walking with Hand Contact. Front. Physiol..

[43] Kochigami K., Jiang J., Kakehashi Y., Au C., Kakiuchi Y., Okada K., Inaba M. (2015). Walking together hand in hand: Design and evaluation of autonomous robot system that a robot recognizes moving direction with a child’s assistance of pulling its hand. Proceedings of the 2015 IEEE/SICE International Symposium on System Integration (SII).

[44] Hieida C., Abe K., Nagai T., Omori T. (2020). Walking hand-in-hand helps relationship building between child and robot. J. Robot. Mechatronics.

[45] Nakane A., Yanokura I., Ichikura A., Okada K., Inaba M. (2023). Development of Robot Guidance System Using Hand-holding with Human and Measurement of Psychological Security. Proceedings of the 2023 32nd IEEE International Conference on Robot and Human Interactive Communication (RO-MAN).

[46] Bönsch A., Hashem D., Ehret J., Kuhlen T.W. (2021). Being Guided or Having Exploratory Freedom: User Preferences of a Virtual Agent’s Behavior in a Museum. Proceedings of the 2021 ACM International Conference on Intelligent Virtual Agents (IVA ’21).

[47] Reinhardt J., Schmidtler J., Körber M., Bengler K. (2016). Follow Me! Wie Roboter Menschen führen sollen. Zeitschrift für Arbeitswissenschaft.

[48] Wakabayashi H., Hiroi Y., Miyawaki K., Ito A. (2023). Path following algorithm with small error for guide robot. Robot Intelligence Technology and Applications 7. RiTA 2022, Gold Coast, Australia, 7–9 December 2022, Lecture Notes in Networks and Systems.

[49] Fujiwara Y., Hiroi Y., Tanaka Y., Ito A. (2023). Development of a Mobile Robot Moving on a Handrail—Control for Preceding a Person Keeping a Distance. Proceedings of the 2015 24th IEEE International Symposium on Robot and Human Interactive Communication (RO-MAN).

[50] Hiroi Y., Ito A., Nakano E. (2009). Evaluation of robot-avatar-based user-familiarity improvement for elderly people. Kansei Eng. Int..

[51] Hiroi Y., Matsunaka S., Ito A. (2012). A mobile robot system with semi-autonomous navigation using simple and robust person following behavior. J. Man, Mach. Technol..

[52] Hiroi Y., Ito A. (2019). A Pedestrian Avoidance Method Considering Personal Space for a Guide Robot. Robotics.

[53] Kerr J., Nickels K. (2012). Robot operating systems: Bridging the gap between human and robot. Proceedings of the 2012 44th Southeastern Symposium on System Theory (SSST).

[54] Cowan N. (2001). The magical number 4 in short-term memory: A reconsideration of mental storage capacity. Behav. Brain Sci..

[55] Belk R. (2021). Ethical issues in service robotics and artificial intelligence. Serv. Ind. J..

[56] Pierce J., Wong R.Y., Merrill N. (2020). Sensor illumination: Exploring design qualities and ethical implications of smart cameras and image/video analytics. Proceedings of the 2020 CHI Conference on Human Factors in Computing Systems.

[57] Luenberger D.G. (1979). Dynamic Systems.

